# 2215. Impact of a Pharmacy-Driven Penicillin Allergy De-Labeling Pilot Program in Pre-Operative Cardiothoracic and Spine Surgery Patients at a Quaternary Hospital

**DOI:** 10.1093/ofid/ofad500.1837

**Published:** 2023-11-27

**Authors:** Hannah Crum, Brandon Gagnon, Krista Gens

**Affiliations:** Southeast Health, Cape Girardeau, Missouri; Abbott Northwestern Hospital, Minneapolis, MN; Abbott Northwestern Hospital, Minneapolis, MN

## Abstract

**Background:**

Studies suggest up to 95% of patients with a reported penicillin (PCN) allergy can tolerate PCNs. Documented PCN allergies are associated with increased risks including Clostridioides difficile infections (CDI) and surgical site infections (SSIs). The goal of this study is to evaluate the effect of a pharmacist-driven PCN allergy de-labeling pilot program on the use of pre-operative beta-lactam antibiotics in cardiothoracic (CT) and spine surgery patients with documented PCN allergies.

**Methods:**

This single-center, quasi-experimental study included adult patients with a charted PCN allergy and admitted for CT or spine surgery in 2021 (control group) and in the 6 months after the intervention pilot began (intervention group, 10/2022 - 03/2023). The primary outcome was rate of beta-lactam use peri-operatively. Secondary outcomes include SSI rates within 30 days after the operation, readmission rates within 30 days, CDI within 30 days of procedure, and hospital length of stay. In the intervention group, qualifying patients were interviewed via phone to assess allergy history. Chart documentation and allergy modification were completed to help ensure optimal antibiotic therapy. Qualified patients were de-labeled or referred to an allergist for outpatient skin testing and/or oral challenge.

**Results:**

There were 214 patients in the control group and 72 in the intervention group. 57 (79.2%) patients in the intervention group were able to complete the interview. A total of 19 patients were referred to an outpatient allergist, and 7 were de-labeled (5 from pharmacist interview alone, 2 after allergist testing). Cefazolin was used pre-operatively in 91 (42.5%) of the control group vs. 55 (76.4%) of the intervention group (p< 0.001). There was no significant difference in SSI rate, 2.3% in the control group vs. 4.2% in the intervention group (p=0.286). No significant difference was noted in any other secondary outcomes except length of stay 3.65 days in the control group vs. 2.06 days in the intervention group (p = 0.0134).

**Conclusion:**

The pharmacy-driven PCN allergy de-labeling pilot program in CT and spine surgery patients was associated with increased beta-lactam use. There was no significant difference in SSI rates; however, the study was underpowered to detect a difference.

**Disclosures:**

**All Authors**: No reported disclosures

